# Detection of Mild COVID-19 in Frail Older Adults Using Simple Inflammatory Indices: A Comparative Cohort Analysis

**DOI:** 10.3390/life15121821

**Published:** 2025-11-27

**Authors:** Yochai Levy, Estela Derazne, Dana Kagansky, Yichayaou Beloosesky, Daniel Trotzky, Miya Sharfman, Nadya Kagansky

**Affiliations:** 1 Shmuel Harofeh Geriatric Hospital, Beer Yaakov 7033001, Israel; batia_nadya.kagansky@moh.gov.il; 2 Gray Faculty of Medicine, Tel Aviv University, Tel Aviv 6997801, Israel; estela.simhoni@gmail.com (E.D.); danak@shamir.gov.il (D.K.); 3 Shamir (Assaf Harofeh) Medical Center, Beer Yaakov 7033001, Israel; 4 Adelson School of Medicine, Ariel University, Ariel 407000, Israel; yjbelo@bezeqint.net; 5 Tel Aviv Sourasky Medical Center (Ichilov), Tel Aviv 6423906, Israel; danieltro@tlvmc.gov.il

**Keywords:** SARS-CoV-2, frailty, geriatric medicine, inflammatory markers, NLR, PLR, COVID-19 diagnostics, hematologic indices

## Abstract

Background: Systemic inflammatory indices such as the neutrophil-to-lymphocyte ratio (NLR) and platelet-to-lymphocyte ratio (PLR) are associated with severe COVID-19, but their role in mild disease among frail older adults remains unclear. Early Israeli admission policies enabled hematologic profiling of asymptomatic and mild cases. Methods: Retrospective cohort of adults ≥65 years admitted to a geriatric center (March 2020–March 2021). Patients with Mild/asymptomatic COVID-19 cases were compared with patients hospitalized for other infections (pneumonia, urinary tract infection, cellulitis). Admission indices such as NLR, derived neutrophil-to-lymphocyte ratio (dNLR), PLR, hemoglobin-to-lymphocyte ratio (HLR), red cell distribution width (RDW), and C-reactive protein (CRP) were analyzed using receiver operating characteristic (ROC) curves. Sensitivity analyses compared COVID-19 with bacterial pneumonia and assessed one-week changes. Results: Among 450 patients (177 COVID-19 and 273 non-COVID; median age 85–86), COVID-19 cases showed lower white blood cell counts (WBC), neutrophils, and CRP but more marked lymphopenia. The most discriminative indices were dNLR, PLR, HLR, and RDW, which differed most (all *p* < 0.001), while NLR and systemic immune-inflammation index (SII) showed limited discrimination. The best AUC was 0.69. dNLR, PLR, and HLR remained elevated after one week. Conclusions: In frail older adults with early or mild COVID-19, modest but consistent hematologic patterns, including lymphopenia with elevated dNLR, PLR, and HLR, and lower RDW, were distinguished COVID-19 from other infections, although single-marker accuracy was limited. These routine indices may assist early differentiation when virologic testing is delayed or unavailable.

## 1. Introduction

In recent years, there has been a global transition toward living alongside COVID-19, with a marked reduction in mortality and hospitalization rates [1]. Nevertheless, SARS-CoV-2 continues to pose a significant medical and epidemiological challenge. New viral variants continue to emerge, and between October 2024 and June 2025, approximately 15 million new cases were reported in the United States, resulting in hundreds of thousands of hospitalizations and tens of thousands of deaths [2].

Older adults, particularly those residing in long-term care and geriatric facilities, remain at the highest risk for severe outcomes and mortality associated with COVID-19 [3]. However, current public health policies no longer mandate routine testing, isolation, or screening in these settings [4,5] and the availability of rapid diagnostic tests and protective equipment has decreased [6]. These changes emphasize the need for improved clinical tools to distinguish COVID-19 and other respiratory infections from common infectious diseases in frail older adults, whose presentations are often atypical and challenging to interpret.

During the initial phase of the COVID-19 pandemic in Israel, widespread outbreaks occurred in nursing homes and long-term care facilities, accounting for a large proportion of national mortality. In response, the Ministry of Health launched the fathers shield—“Magen Avot” program [7]. Long-term care facilities implemented surveillance testing, and positive cases were isolated, often in designated COVID-19 wards within geriatric hospitals. These wards admitted older adults with mild or asymptomatic infections who could not be isolated at home or required continuous medical care [8]. This unique setting provided a rare opportunity to evaluate numerous frail older adults during the initial stages of COVID-19, including older adults who might not have been hospitalized under usual clinical circumstances.

Routine laboratory parameters such as neutrophil, lymphocyte, and platelet counts, alongside C-reactive protein (CRP), are commonly used as markers of systemic inflammation [9,10]. Indices derived from these values, including neutrophil-to-lymphocyte ratio (NLR), platelet-to-lymphocyte ratio (PLR), derived NLR (dNLR), monocyte-to-lymphocyte ratio (MLR), lymphocyte-to-monocyte ratio (LMR), systemic immune-inflammation index (SII), red cell distribution width (RDW), hemoglobin-to-lymphocyte ratio (HLR), and hemoglobin-to-platelet ratio (HPR), have been studied as markers of inflammation in infections, malignancies, and cardiovascular disease [11,12,13].

Extensive research has investigated these biomarkers in severe COVID-19 [14,15,16,17,18,19]. However, their behavior in mild disease, particularly among vulnerable geriatric populations, remains poorly understood. Furthermore, limited data are available comparing these markers between mild COVID-19 cases and other infections, particularly among older adults. In two previous studies, we demonstrated that inflammatory markers such as NLR and PLR appeared to be influenced more by age and frailty than by the severity of COVID-19 [20,21], despite the widely reported association of COVID-19 with a hyperinflammatory response [22].

The present study extends this work by examining whether early, mild COVID-19 induces a measurable inflammatory response compared with other common infections leading to hospitalization in frail older adults. Specifically, we aimed to compare inflammatory markers, including NLR, PLR, dNLR, MLR, LMR, SII, RDW, HLR, HPR, and CRP, between older adults hospitalized with mild COVID-19 and those admitted with other infections. We also evaluated whether these indices could aid in differentiating early-stage COVID-19 from non-COVID-19 infections in this population.

## 2. Materials & Methods

### 2.1. Study Design and Population

This was an observational comparative cohort study conducted at a 340-bed geriatric hospital (GH). At the onset of the COVID-19 pandemic in Israel, and in accordance with government regulations, the hospital established a designated COVID-19 department. The GH is affiliated with a nearby tertiary hospital and receives patients directly from its emergency department, which is then transferred to three acute geriatric departments.

Routine blood tests are obtained for all acute admissions within 24 h of hospitalization and subsequently performed as clinically indicated during the remainder of the stay. Blood count results were obtained using the Sysmex XN-500 instrument (Sysmex Corporation, Kobe, Japan). Neutrophils, lymphocytes and monocytes using flow cytometry, platelets using DC Impedance method with hydrodynamic focusing method and hemoglobin using the SLS (sodium lauryl sulphate) method. CRP was tested with photometric assays on Cobas 6000 analyser (Roche Diagnostics, Mannheim, Germany).

**Figure A1:**
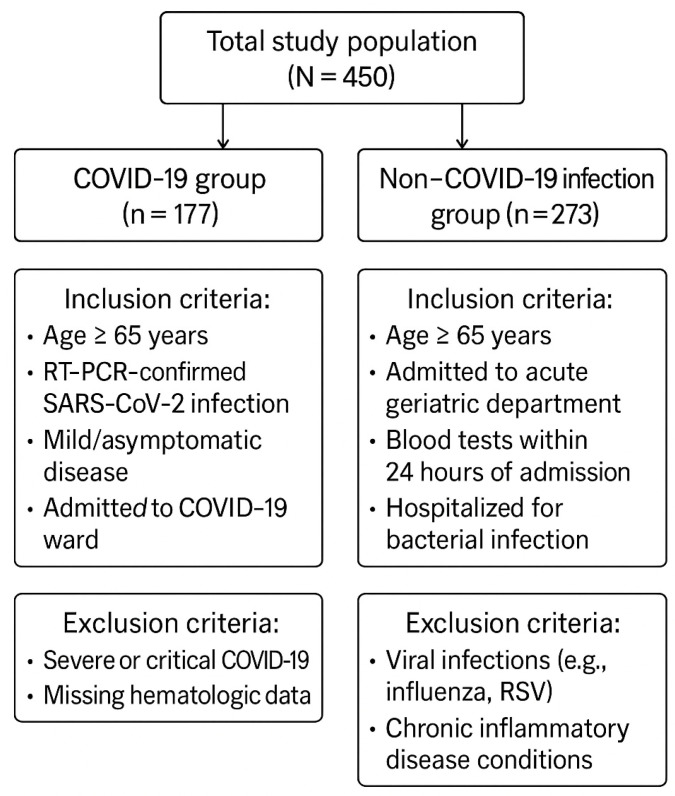
Study population flowchart. Abbreviations: CRP, C-reactive protein; RSV, respiratory syncytial virus; SARS-CoV-2, severe acute respiratory syndrome coronavirus 2.

Two cohorts were included (Appendix A Figure A1). The first comprised patients who tested positive for SARS-CoV-2 with mild or asymptomatic disease and were admitted to the designated COVID-19 departments. Most patients in the COVID-19 group remained asymptomatic or experienced only mild disease throughout hospitalization, a pattern previously demonstrated in a prior publication on the same cohort [18]. The second cohort included patients admitted to the acute geriatric departments with suspected bacterial infections, such as pneumonia, urinary tract infection (UTI), or cellulitis. Data collection covered the period during which the designated COVID-19 departments were active, from March 2020 to March 2021. SARS-CoV-2 testing was performed using real-time reverse transcription polymerase chain reaction (RT-PCR) on throat swabs.

Inclusion criteria were admission to either a designated COVID-19 department or an acute geriatric department for a primary diagnosis of another infectious cause. Data were retrieved from electronic medical records (EMRs) and included demographic variables, comorbidities, medications, laboratory results, and clinical symptoms.

In the pneumonia subgroup, 150 patients were included in the baseline analyses, encompassing those with a primary diagnosis of pneumonia (*n* = 144) and an additional six patients with secondary sepsis attributed to pneumonia.

### 2.2. Inflammatory Indices

Peripheral blood–derived inflammatory indices were calculated based on routine laboratory parameters. The neutrophil-to-lymphocyte ratio (NLR) was defined as the absolute neutrophil count divided by the lymphocyte count and has been widely studied as a marker of systemic inflammation and infection, including pneumonia, urinary tract infection, and COVID-19 [23,24,25]. The derived neutrophil-to-lymphocyte ratio (dNLR) was calculated as neutrophils divided by (total leukocytes minus lymphocytes) and has been shown to predict outcomes in infectious diseases such as sepsis and COVID-19 [26,27]. The platelet-to-lymphocyte ratio (PLR) was defined as platelet count divided by lymphocytes and reflects inflammatory burden in bacterial pneumonia and COVID-19 [28,29]. The monocyte-to-lymphocyte ratio (MLR) and its inverse, the lymphocyte-to-monocyte ratio (LMR), have been investigated as indicators of immune activation in bacterial infections, tuberculosis, and, more recently, COVID-19 [30,31]. The systemic immune-inflammation index (SII) was calculated as (platelets × neutrophils) ÷ lymphocytes [32] and has been correlated with disease severity in community-acquired pneumonia and COVID-19 [33]. Red cell distribution width (RDW), reflecting anisocytosis, was also assessed as a prognostic biomarker in sepsis and COVID-19 [34,35]. In addition, we evaluated the hemoglobin-to-lymphocyte ratio (HLR) [36] and hemoglobin-to-platelet ratio (HPR) [36], which have recently been proposed as markers of systemic inflammation with emerging prognostic relevance in infectious and inflammatory conditions, including COVID-19.

### 2.3. Statistical Analysis

Sample size was calculated using the Universität Wien calculator (https://homepage.univie.ac.at/robin.ristl/samplesize.php?test=wilcoxon), according to Noether (1987)( accessed on 21 February 2025), to compare two groups for inflammatory markers that were not normally distributed (including NLR, PLR, dNLR, MLR, LMR, SII, RDW, HLR, HPR, and CRP). For a two-sided alpha = 0.05, power = 0.8, and *p* (X > Y) = 0.65, the minimum sample size required per group was 59.

The mean age between the two groups was compared using the *t*-test. Categorical variables were compared using the chi-squared test (or Fisher’s exact test in the case of 2 × 2 tables). Ordinal variables (total number of diseases, total number of medications) or interval variables that were not normally distributed (inflammatory markers and blood test results) were compared using the Mann–Whitney test. Receiver operating characteristic (ROC) curves and the area under the curve (AUC) were calculated to evaluate the classification power of the markers. COVID-19 and non-COVID-19 infection patients were compared in the main analysis, and a sub-analysis compared COVID-19 patients with those with bacterial pneumonia. Statistical analysis was performed using IBM SPSS Statistics for Windows, Version 29.0 (Armonk, NY, USA). A two-sided *p*-value ≤ 0.05 was considered statistically significant.

## 3. Results

A total of 450 patients were included in the analysis, comprising 177 patients admitted to a COVID-19-designated department and 273 patients with presumed bacterial infections admitted to acute geriatric departments at a geriatric medical center between March 2020 and March 2021. Among the non-COVID-19 infection (NCI) group, the most common diagnosis was pneumonia (*n* = 144, 52.7%), followed by urinary tract infection (UTI) (*n* = 79, 28.9%) and cellulitis (*n* = 39, 14.3%). Less common diagnoses included sepsis and other non-specific bacterial infections.

Baseline characteristics are described in Table 1. The median age was similar between groups (COVID-19: 85 [IQR 77–91] vs. NCI: 86 [IQR 81–91], *p* = 0.068). Most patients were female (~61%), and about 55% were widowed in both groups. However, COVID-19 patients were more likely to reside in nursing homes (53.4% vs. 17.9%, *p* < 0.001).

Comorbidity burden was substantial across both groups, with over 80% of patients presenting with five or more chronic diseases (80.8% in the COVID-19 group vs. 83.5% in the NCI group; *p* = 0.013). However, several specific comorbidities were more prevalent in the NCI group, including congestive heart failure (45.1% vs. 22.6%; *p* < 0.001), chronic renal failure (39.9% vs. 22.0%; *p* < 0.001), dementia (52.0% vs. 34.5%; *p* < 0.001), depression (25.6% vs. 15.8%; *p* = 0.014), asthma or chronic obstructive pulmonary disease (30.1% vs. 9.0%; *p* < 0.001), and coronary artery disease (43.6% vs. 29.9%; *p* = 0.004). The proportion of patients treated with more than six chronic medications was significantly higher in the COVID-19 group (71.2% vs. 60.4%; *p* = 0.002). In terms of medication use, patients with COVID-19 were less likely to receive ACE inhibitors or ARBs (43.5% vs. 61.5%; *p* < 0.001), beta-blockers (47.5% vs. 58.6%; *p* = 0.026), and insulin (19.8% vs. 36.3%; *p* < 0.001), but more likely to be treated with vitamin D (36.2% vs. 20.1%; *p* < 0.001), Eltroxin (20.9% vs. 9.5%; *p* < 0.001), antipsychotics (36.2% vs. 10.3%; *p* < 0.001), and antidepressants (28.2% vs. 41.8%; *p* = 0.004).

Presenting symptoms differed significantly between the groups (Table 2). Most COVID-19 patients were asymptomatic at admission (64.4% vs. 16.8%, *p* < 0.001). Classic infectious symptoms such as fatigue (4.0% vs. 44.7%), headache (0.0% vs. 15.4%), fever (10.7% vs. 26.4%), and cough (7.9% vs. 25.3%) were markedly less common in the COVID-19 group (all *p* < 0.001). Dyspnea was also less frequent (24.9% vs. 38.8%, *p* = 0.002).

Laboratory findings at admission revealed significant differences between the groups (Table 3). COVID-19 patients had lower white blood cell counts compared to those with non-COVID-19 infections (median 7.8 vs. 9.9 × 10^3^/µL, *p* < 0.001), along with lower neutrophil percentages (72.0% vs. 76.4%, *p* < 0.001) and absolute neutrophil counts (5.53 vs. 6.09 × 10^3^/µL, *p* = 0.004). Lymphocyte percentages were similar between groups (*p* = 0.606), but absolute lymphocyte counts were lower in the COVID-19 group (1.29 vs. 1.6 × 10^3^/µL, *p* < 0.001). Red cell distribution width (RDW) was significantly lower in COVID-19 patients (14.4% vs. 15.8%, *p* < 0.001), while hemoglobin levels were slightly higher (12.3 vs. 11.8 g/dL, *p* = 0.044). Platelet counts and albumin levels did not differ significantly. Importantly, C-reactive protein (CRP), a key inflammatory marker, was markedly lower in the COVID-19 group (26.0 vs. 47.8 mg/dL, *p* < 0.001), suggesting a milder inflammatory response at presentation. No significant differences were found in creatinine, urea, or lactate dehydrogenase (LDH) levels.

Inflammatory and hematologic index markers showed several notable differences between groups (Table 4). The derived neutrophil-to-lymphocyte ratio (dNLR) was significantly higher in COVID-19 patients (median 2.51 vs. 1.69, *p* < 0.001), as was the platelet-to-lymphocyte ratio (PLR; 191.1 vs. 150.0, *p* < 0.001). Hemoglobin-to-lymphocyte ratio (HLR) was also elevated in COVID-19 patients (9.50 vs. 7.49, *p* < 0.001). In contrast, traditional neutrophil-to-lymphocyte ratio (NLR), monocyte-to-lymphocyte ratio (MLR), lymphocyte-to-monocyte ratio (LMR), systemic immune-inflammation index (SII), and hemoglobin-to-platelet ratio (HPR) did not differ significantly between groups.

**Figure 1:**
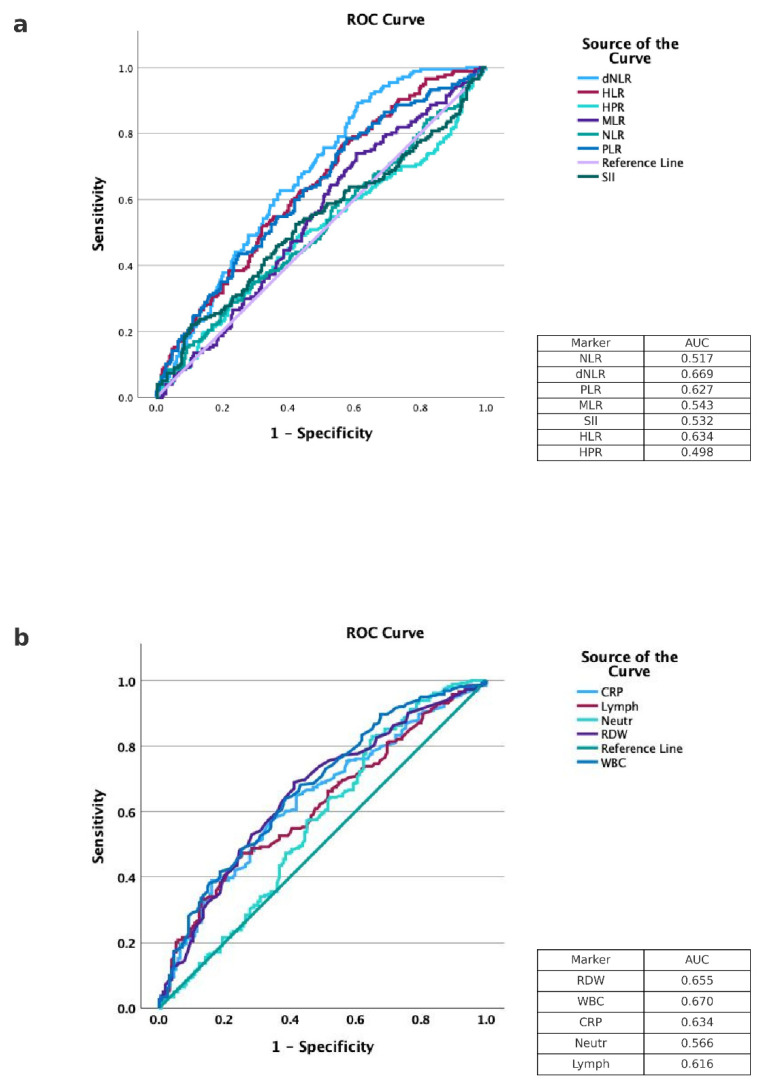
Receiver operating characteristic (ROC) curves of inflammatory markers differentiating COVID-19 from non–COVID-19 infections. (**a**) Receiver operating characteristic (ROC) curves for derived inflammatory ratios: neutrophil-to-lymphocyte ratio (NLR), derived NLR (dNLR), platelet-to-lymphocyte ratio (PLR), monocyte-to-lymphocyte ratio (MLR), systemic immune-inflammation index (SII), hemoglobin-to-lymphocyte ratio (HLR), and hemoglobin-to-platelet ratio (HPR). (**b**) ROC curves for conventional hematologic and inflammatory markers, including red cell distribution width (RDW), white blood cell count (WBC), C-reactive protein (CRP), neutrophil count, and lymphocyte count. Corresponding area under the curve (AUC) values are presented in the in-plot tables.

To evaluate the predictive performance of laboratory parameters, ROC curves were constructed both for direct measures (such as total leukocyte count, neutrophils, lymphocytes, and platelets) and for derived inflammatory indices combining several parameters, including NLR, PLR, and others. Overall, discrimination between patients with COVID-19 and those hospitalized with other infectious diseases is presented in Figure 1, with the highest AUC reaching 0.69. To validate the robustness of these findings, we performed two sensitivity analyses. First, we examined whether the timing of blood tests influenced the inflammatory profile in COVID-19 patients, given that many were admitted shortly after a positive screening PCR while still pre-symptomatic. A comparison was made between inflammatory markers measured one week after admission in COVID-19 patients and baseline values in NCI patients (Table 5). At one week, COVID-19 patients demonstrated significantly higher values in several markers, including dNLR (median 2.80 vs. 1.69, *p* < 0.001), PLR (210.5 vs. 150.0, *p* < 0.001), SII (1124 vs. 926.8, *p* = 0.021), and HLR (8.56 vs. 7.49, *p* = 0.009). RDW remained significantly lower in COVID-19 patients even after one week (14.80 vs. 15.80, *p* = 0.001). HPR values were slightly but significantly lower in the COVID-19 group (0.04 vs. 0.05, *p* = 0.022). NLR showed a trend toward higher levels in COVID-19 patients but did not reach statistical significance (*p* = 0.055). No significant differences were observed in the MLR.

Second, we compared COVID-19 patients exclusively to those with suspected bacterial pneumonia (*n* = 144), the most clinically similar and prevalent infection in the NCI group. The total pneumonia cohort included 150 patients, encompassing 144 with a primary diagnosis of pneumonia and six additional patients with sepsis secondary to pneumonia. Data for this cohort are presented in Appendix A Table A1 (baseline characteristics) and Appendix A Table A2 (inflammatory marker results). Baseline characteristics of pneumonia patients were broadly similar to those of the overall NCI population, and the inflammatory markers results mirrored those of the primary analysis (Appendix A Table A2).

## 4. Discussion

The present study examined systemic inflammatory markers in frail older adults with asymptomatic or mild COVID-19, comparing them to older patients hospitalized with other common infectious diseases. Although extensive research has investigated inflammation biomarkers such as NLR in severe COVID-19 [15,17,19,25,27,37], their behavior in mild disease, particularly among vulnerable geriatric populations, remains insufficiently characterized. During the early stages of the pandemic, Israel’s public health policy mandated universal screening and isolation following any suspected exposure to SARS-CoV-2, including in nursing homes. This policy created a unique opportunity to evaluate hematologic responses in infected older adults at an unusually early stage, frequently preceding symptom onset, and to assess differences from other infectious illnesses in a frail, multimorbid cohort.

In a previous study [20,21], we demonstrated that age and frailty exerted a stronger influence on inflammatory ratios such as NLR and PLR than the diagnosis of COVID-19 itself, with NLR showing the most marked variations. We also found that although most COVID-19 patients admitted to our geriatric center were frail, they typically experienced only mild disease, and mortality was minimal. Building on these findings, the current study assessed whether composite blood-derived measures could differentiate between mild or asymptomatic COVID-19 and other common infections in frail older patients. Given the systemic inflammatory response described in severe COVID-19, we hypothesized that these alterations might also appear in mild disease, even in the absence of clinical severity.

Although the groups were similar in age and sex, differences in comorbidities and medications were evident. Many COVID-19 patients came from long-term care facilities, where documentation and treatment patterns differed. Nonetheless, both groups were composed mainly of frail, multimorbid old adults with conditions such as dementia and cardiovascular disease. Unlike our earlier comparative study between general and geriatric hospitals, no independent age effect was found, probably due to the uniformly high age and complexity in both groups.

Neither group showed substantial leukocytosis. Bacterial infections were associated with slightly higher WBC, but overall values remained modest. This is consistent with the blunted inflammatory response typical in older adults. Immunosenescence, characterized by reduced neutrophil proliferation, lower cytokine release, and a tendency toward lymphopenia rather than leukocytosis, has been recognized as a hallmark of infection in older adults [38,39]. Thus, the absence of marked leukocytosis, even in serious bacterial infections, aligns with the expected immune profile of this population and should be considered when assessing infection severity in older adults.

Lymphopenia is common in both aging [40] and viral illnesses [41], including influenza and HIV. In COVID-19, lymphocyte depletion has been especially prominent, affecting over 60% of hospitalized patients [42], and correlate with worse outcomes [43]. In contrast, bacterial infections typically elicit neutrophilic leukocytosis, although lymphopenia may also appear as a prognostic marker [44]. Our results are consistent with this pattern: neutrophil counts were higher in bacterial infections, while lymphopenia was more pronounced in COVID-19 patients, even among asymptomatic or mild disease patients. This suggests that early lymphocyte depletion could indicate viral etiology in the older adults.

Monocytes counts did not increase in COVID-19, consistent with reports showing that COVID-19 alters monocyte function rather than number. Severe cases show reduced HLA-DR expression and expansion of pro-inflammatory subsets, but such changes are not necessarily reflected in mild disease.

Red cell indices provided additional insights. Hemoglobin levels were slightly higher in COVID-19 patients, a difference that reached statistical significance. This may reflect greater anemia prevalence in those with other infections or, a milder inflammatory burden in COVID-19, supported by lower CRP levels. The RDW was significantly lower in the COVID-19 group, even among asymptomatic cases. RDW is recognized as a prognostic biomarker in sepsis and COVID-19 [34,35,45] yet we did not find a significant affect in mild COVID-19 patients.

Derived ratios and composite indices showed mixed findings. NLR, though widely studied in COVID-19, did not differentiate COVID-19 from other infections. In contrast, the dNLR was consistently higher in COVID-19 patients and remained elevated at follow-up. PLR was also significantly higher and remained elevated after one week. This finding aligns with studies linking increased PLR to poor outcomes in pneumonia [23], sepsis [29], and COVID-19 [15,28]. MLR did not differ at baseline but increased significantly in bacterial infections during follow-up, likely reflecting late-phase monocytosis, and with declining neutrophil counts [46,47].

The systemic immune-inflammation index (SII), which integrates neutrophils, lymphocytes, and platelets, was consistently higher in COVID-19 patients at both time points. Although some studies suggest that SII may outperform NLR in prognostic accuracy [18,48] In our cohort, it did not demonstrate clear diagnostic separation. Although SII was statistically higher in COVID-19 patients, its diagnostic performance was limited, as demonstrated by a relatively low ROC score compared to other more commonly used markers. The hemoglobin-to-lymphocyte ratio (HLR), which has been less extensively studied in infectious diseases, was also higher in COVID-19 patients, likely reflecting the combination of slightly higher hemoglobin levels and lower lymphocyte counts.

Taken together, these findings suggest that several hematologic measures, including dNLR, PLR, MLR, SII, and HLR-differed between mild COVID-19 and other infections. Their diagnosis suggests potential diagnostic value in frail older patients. The most consistent discriminating features were WBC, CRP, lymphopenia, PLR, Dnlr, and SII.

This study has several limitations. First, it was retrospective and a single center, which may restrict the generalizability. Nevertheless, the Israeli policy of admitting all COVID-19 patients, including asymptomatic ones, provided a unique view of early disease, which is rarely represented in the literature. The study population was heterogeneous with respect to comorbidities, although both groups consisted of frail older adults with a high comorbidity burden.

Since the markers were not normally distributed and do not have established cutoff values that would allow dichotomization for logistic regression, we did not perform a multivariable analysis. In addition, there was multicollinearity among independent variables. To mitigate this, we performed prespecified sensitivity analyses (including one-week follow-up values and a pneumonia-only subgroup) and interpreted the findings conservatively, considering biological plausibility and prior literature. Nevertheless, these results should be regarded as hypothesis-generating. Confirmation with larger, prospectively designed cohorts with multivariable adjustment and multiplicity control is warranted.

Additional limitations can be noted, including the absence of a healthy control group, as all participants were acutely ill older adults hospitalized in geriatric departments, with no healthy cohort was available for comparison within this clinical setting. Data on vitamin D, PSA, and peripheral blood smears were unavailable, as these tests are not routinely performed during acute admissions. Similarly, definitive microbiological confirmation (e.g., sputum cultures) was not obtained, since such tests have limited diagnostic yield in older adults and rarely influence treatment decisions. These limitations underscore the need for future, ideally prospective, studies incorporating standardized biomarker assessment and microbiological verification in well-characterized geriatric populations. Finally, patients with severe COVID-19 were excluded, and therefore, the conclusions cannot be applied to advanced disease.

## 5. Conclusions

In frail older adults with mild or asymptomatic SARS-CoV-2 infection, lymphopenia, an elevated PLR, an increased dNLR, and low RDW were the most consistent distinguishing hematologic features. The SII was also consistently higher in COVID-19 than in non-COVID-19 comparators. These findings suggest that, while careful clinical assessment and virological testing remain essential, these readily available hematologic parameters can assist early differentiation when testing is delayed or impractical.

## Figures and Tables

**Table 1:** Baseline demographic and clinical characteristics of older adults hospitalized with COVID-19 and non–COVID-19 infections.

	COVID-19		Non-COVID-19 Infection		
	N	Median (25th–75th)	N	Median (25th–75th)	*p*
Age (years, median) (IQR)	177	85 (77–91)	273	86 (81–91)	0.068
	N	%	N	%	
Sex					0.921
Male	68	38.4	107	39.2	
Female	109	61.6	166	60.8	
Residence					<0.001
Home	82	46.6	224	82.1	
Nursing Home	94	53.4	49	17.9	
Total number of diseases					0.013
1	1	0.6	1	0.4	
2	3	1.7	6	2.2	
3	14	7.9	13	4.8	
4	16	9	25	9.2	
≥5	143	80.8	228	83.5	
Comorbidities					
CHF	40	22.6	123	45.1	<0.001
CRF	39	22	109	39.9	<0.001
Dementia	61	34.5	142	52.0	<0.001
Depression	28	15.8	70	25.6	0.014
Asthma COPD	16	9	82	30.1	<0.001
CVA	38	21.5	66	24.2	0.567
DM	87	49.2	134	49.1	1.000
HTN	143	80.8	233	85.3	0.241
Coronary Disease	53	29.9	119	43.6	0.004
Hyperlipidemia	75	42.4	113	41.4	0.845
Total number of medications					0.002
0	0	0	1	0.4	
1–3	8	4.5	41	15.0	
4–6	43	24.3	66	24.2	
>6	126	71.2	165	60.4	
ACE/ARB	77	43.5	168	61.5	<0.001
B-blockers	84	47.5	160	58.6	0.026
Insulin	35	19.8	99	36.3	<0.001
CC-blockers	63	35.6	117	42.9	0.140
Vit.D	64	36.2	55	20.1	<0.001
Antiplatelets	56	31.6	81	29.7	0.676
Eltroxin	37	20.9	26	9.5	<0.001
Anticoagulants	77	43.5	98	35.9	0.114
Antipsychotics	64	36.2	28	10.3	<0.001
Antidepressants	50	28.2	114	41.8	0.004

Abbreviations: ACE, angiotensin-converting enzyme; ARB, angiotensin II receptor blocker; B-blockers, Beta-blockers; CC-blockers, calcium-channel blocker; CHF, congestive heart failure; COPD, chronic obstructive pulmonary disease; CRF, chronic renal failure; CVA, cerebrovascular accident; DM, diabetes mellitus; HTN, hypertension; IQR, interquartile range; Eltroxin, levothyroxine.

**Table 2:** Symptom distribution among older adults hospitalized with COVID-19 and non–COVID-19 infections.

	COVID-19		Non-COVID-19 Infection		
	N	%	N	%	*p*
Anosmia	0	0.0	3	1.1	0.283
Diarrhea	1	0.6	8	2.9	0.096
Fatigue	7	4.0	122	44.7	<0.001
Headache	0	0.0	42	15.4	<0.001
Fever	19	10.7	72	26.4	<0.001
Cough	14	7.9	69	25.3	<0.001
Anxiety	1	0.6	6	2.2	0.253
Delirium	2	1.1	17	6.2	0.008
Dyspnea	44	24.9	106	38.8	0.002
Syncope	1	0.6	3	1.1	1.000
Instability	25	14.1	0	0.0	<0.001
Chest pain	2	1.1	9	3.3	0.214
Change of appetite	3	1.7	21	7.7	0.005
No symptoms	114	64.4	46	16.8	<0.001

**Table 3:** Laboratory findings among older adults hospitalized with COVID-19 and non–COVID-19 infections.

		COVID-19		Non-COVID-19 Infection		*p*
		N	Median (25th–75th)	N	Median (25th–75th)	
WBC	10^3^/µL	177	7.8 (5.7–10.1)	273	9.9 (7.7–13.9)	<0.001
Neutr %	%	177	72 (62.8–80.6)	273	76.4 (69.0–82.8)	<0.001
Neutr	10^3^/µL	177	5.53 (3.73–7.91)	273	6.09 (4.92–7.98)	0.004
Lymph%	%	177	17.9 (10.9–25.8)	273	17.3 (11.2–23.8)	0.606
Lymph	10^3^/µL	177	1.29 (0.9–1.7)	273	1.6 (1.1–2.2)	<0.001
Monocytes	10^3^/µL	177	0.6 (0.4–0.8)	273	0.7 (0.5–0.9)	0.002
PLT	10^3^/µL	177	239 (194–324)	273	241 (189–306)	0.349
RDW	%	177	14.4 (13.4–15.9)	271	15.8 (14.3–17.3)	<0.001
Hb	g/dL	177	12.3 (10.6–13.6)	272	11.8 (10.7–12.9)	0.044
Albumin	g/dL	171	3.69 (3.34–4.06)	271	3.71 (3.37–3.95)	0.417
CRP	mg/L	155	26 (11–56)	266	47.8 (21.7–89.6)	<0.001
Creatinine	mg/dL	176	0.92 (0.72–1.2)	268	0.91 (0.72–1.35)	0.626
LDH	U/L	118	334 (272–416)	241	345.0 (292–419)	0.251
Urea	mg/dL	176	50 (35–74.5)	261	50.8 (36.0–67.3)	0.531

Abbreviations: WBC, white blood cell count; Neutr, neutrophil count; Lymph, lymphocyte count; PLT, platelet count; RDW, red cell distribution width; Hb, hemoglobin; CRP, C-reactive protein; LDH, lactate dehydrogenase; Urea, blood urea nitrogen; IQR, interquartile range.

**Table 4:** Inflammatory and hematologic index markers among older adults hospitalized with COVID-19 and non–COVID-19 infections.

		COVID-19	Non-COVID Infection		*p*
	N	Median (25th–75th)	N	Median (25th–75th)	
NLR	177	3.95 (2.44–7.36)	273	3.89 (2.52–6.58)	0.512
dNLR	177	2.51 (1.67–4.15)	273	1.69 (0.78–2.85)	<0.001
PLR	177	191.12 (137.1–282.2)	273	150 (99.5–222.6)	<0.001
MLR	177	0.46 (0.34–0.67)	273	0.44 (0.29–0.64)	0.120
LMR	177	2.16 (1.50–2.93)	273	2.29 (1.55–3.42)	0.120
SII	177	1068 (523–1946)	273	927 (542–1717)	0.237
HLR	177	9.50 (6.78–13.60)	272	7.49 (5.02–11.28)	<0.001
HPR	177	0.05 (0.03–0.07)	272	0.05 (0.04–0.06)	0.939

Abbreviations: NLR, neutrophil-to-lymphocyte ratio; dNLR, derived neutrophil-to-lymphocyte ratio; PLR, platelet-to-lymphocyte ratio; MLR, monocyte-to-lymphocyte ratio; LMR, lymphocyte-to-monocyte ratio; SII, systemic immune-inflammation index; HLR, hemoglobin-to-lymphocyte ratio; HPR, hemoglobin-to-platelet ratio; IQR, interquartile range.

**Table 5:** Inflammatory and hematologic markers in COVID-19 patients one week after admission compared with non–COVID-19 infection patients at admission.

	COVID-19 One Week After Admission		Non-COVID Infection at Admission		*p*
	N	Median (25th–75th)	N	Median (25th–75th)	
NLR	100	4.57 (2.87–7.53)	273	3.98 (2.52–6.58)	0.055
dNLR	100	2.80 (1.99–4.30)	273	1.69 (0.78–2.85)	<0.001
PLR	95	210.5 (137.0–275.0)	273	150.0 (99.5–222.6)	<0.001
MLR	100	0.47 (0.32–0.66)	273	0.44 (0.29–0.64)	0.288
LMR	100	2.11 (1.51–3.13)	273	2.29 (1.55–3.42)	0.288
SII	95	1124.0 (724.1–1846.1)	273	926.8 (541.9–1717.2)	0.021
RDW (%)	100	14.80 (13.65–16.20)	271	15.80 (14.30–17.30)	0.001
HLR	100	8.56 (6.36–12.20)	272	7.49 (5.02–11.28)	0.009
HPR	95	0.04 (0.03–0.06)	272	0.05 (0.04–0.06)	0.022

Abbreviations: NLR, neutrophil-to-lymphocyte ratio; dNLR, derived neutrophil-to-lymphocyte ratio; PLR, platelet-to-lymphocyte ratio; MLR, monocyte-to-lymphocyte ratio; LMR, lymphocyte-to-monocyte ratio; SII, systemic immune-inflammation index; RDW, red cell distribution width; HLR, hemoglobin-to-lymphocyte ratio; HPR, hemoglobin-to-platelet ratio.

## Data Availability

The data presented in this study are available on reasonable request from the corresponding author. The data are not publicly available due to institutional privacy regulations.

## Appendix A

**Table A1:** Baseline demographic and clinical characteristics of older adults hospitalized with COVID-19 and bacterial pneumonia.

	COVID-19		Bacterial Pneumonia		
	N	Median (25th–75th)	N	Median (25th–75th)	*p*
Age	177	85 (77–91)	150	87 (81–91)	0.041 #
	N	%	N	%	
Sex					0.057
Male	68	38.4	74	49.3	
Female	109	61.6	76	50.7	
Residence					0.194
Home	82	46.6	117	78.0	
Nursing Home	94	53.4	33	22.0	
Total number of diseases					0.009 *
1	1	0.6	1	0.7	
2	3	1.7	6	4.0	
3	14	7.9	4	2.7	
4	16	9	11	7.3	
≥5	143	80.8	128	85.3	
Total number of medications					0.007 *
0	0	0	1	0.7	
1–3	8	4.5	24	16.0	
4–6	43	24.3	35	23.3	
>6	126	71.2	90	60.0	

Abbreviations: CHF, congestive heart failure; CRF, chronic renal failure; CVA, cerebrovascular accident; DM, diabetes mellitus; HTN, hypertension. Statistical tests: * Mann–Whitney test; # *t*-test; Fisher’s exact probability test for categorical variables.

**Table A2:** Inflammatory and hematologic markers in older adults hospitalized with COVID-19 and bacterial pneumonia.

	COVID-19 (N = 177)	Bacterial Pneumonia (N = 150)	*p*
	Median (25th–75th)	Median (25th–75th)	
NLR	3.95 (2.44–7.36)	4.14 (2.76–6.58)	0.862
dNLR	2.51 (1.67–4.15)	1.68 (0.78–2.72)	<0.001
PLR	191.1 (137.1–282.2)	159.6 (107.4–227.4)	0.001
MLR	0.46 (0.34–0.67)	0.45 (0.31–0.64)	0.460
LMR	2.16 (1.50–2.93)	2.24 (1.55–3.19)	0.460
SII	1068 (523–1946)	959 (579–1726)	0.612
RDW	14.4 (13.4–15.9)	15.7 (14.4–17.0)	<0.001
HLR	9.50 (6.78–13.60)	7.83 (5.58–11.49)	0.001
HPR	0.05 (0.03–0.07)	0.05 (0.04–0.07)	0.600

Abbreviations: NLR, neutrophil-to-lymphocyte ratio; dNLR, derived neutrophil-to-lymphocyte ratio; PLR, platelet-to-lymphocyte ratio; MLR, monocyte-to-lymphocyte ratio; LMR, lymphocyte-to-monocyte ratio; SII, systemic immune-inflammation index; RDW, red cell distribution width; HLR, hemoglobin-to-lymphocyte ratio; HPR, hemoglobin-to-platelet ratio. Note: The pneumonia cohort (*n* = 150) includes patients with a primary diagnosis of pneumonia (*n* = 144) and six additional patients with secondary sepsis attributed to pneumonia.
